# Drill choice as a modifiable factor in pediatric sEEG safety

**DOI:** 10.1007/s00381-026-07319-4

**Published:** 2026-06-20

**Authors:** Mahalia Dalmage, Sunny Abdelmageed, Lucinda Chiu, Oludamilola Adeshina, Emma Poland, Jeffrey S. Raskin

**Affiliations:** 1 Division of Biological Sciences, University of Chicago Pritzker School of Medicine, Chicago, IL USA; 2 Division of Pediatric Neurosurgery, Ann and Robert H. Lurie Children’s Hospital, Chicago, IL USA; 3 Department of Neurosurgery, Northwestern University Feinberg School of Medicine, Chicago, IL USA; 4 Department of Neurological Surgery, Rush University Medical Center, Chicago, IL USA; 5 Marshall University Joan C. Edwards School of Medicine, Huntington, WV USA

**Keywords:** Burr holes, Drug resistant epilepsy, Patient care, Quality improvement, Patient Safety, SEEG Complications, Surgical Technology, Thermal Injury

## Abstract

**Purpose:**

Stereoelectroencephalography (sEEG) procedures require drilling serial burr holes for electrode placement. Key drilling characteristics include haptic feedback, ease of use, and time-efficient burr hole creation. Orthopedic drill bits are often repurposed for sEEG. We present a case series highlighting sEEG as an application for a new-to-market neurosurgery-tailored drill.

**Methods:**

We performed a retrospective chart review on patients who underwent sEEG at our institution from 2022–2023. Patients were grouped by type of drill, Phasor^®^ (a disposable battery-operated drill 3.20-mm diameter, 210-mm usable length) or Stryker^®^ (Orthopedic Drill Cordless).

**Results:**

Twelve patients underwent sEEG with the neurosurgery-tailored drill (2 unilateral and 10 bilateral); on average 10.7 ± 1.6 electrodes were placed per patient and mean operative duration was 162.9 ± 49 min. Tactile feel of bony layers and consistent drill time per burr hole (< 15 s) was subjectively noted. No complications were noted. Five patients underwent sEEG with the Stryker^®^ orthopedic drill (2 unilateral and 3 bilateral); an average of 11 ± 2.9 electrodes were placed per patient, mean operative duration was 176.4 ± 18.9 min. Subjectively, drill time per burr hole was variable and increased in duration between the first and final hole. One patient incurred a skin burn at the drill site which led to wound dehiscence and infection requiring prolonged wound care and antibiotics.

**Conclusion:**

The Phasor^®^ drill offers a lightweight, reliable, consistent, safe and efficient option for sEEG burr hole creation and may be considered as an alternative to the Stryker^®^ orthopedic drill.

## Introduction

Stereoelectroencephalography (sEEG) is a procedure used to identify the epileptogenic zone in patients with drug-resistant epilepsy (DRE) and elucidate any overlap of eloquent cortex and is recommended by the International League Against Epilepsy (ILAE) when noninvasive data is discordant [1, 2]. SEEG facilitates pathophysiologic network identification via the placement of intraparenchymal depth electrodes through small burr holes [3, 4]. SEEG is relatively safe, with a pooled complication rate of 1.3% for sEEG insertion and monitoring [5]. Although rare, complications occur from surgical (e.g. hemorrhage, infection, stroke) and hardware (e.g. bolt migration, electrode breakage) factors. Complications can be minimized but not eliminated. Hardware-related complications are rare; superficial wound infections were reported in 1.4% of procedures and implant malfunction, including accidental electrode dislodging, in 0.4% [5]. SEEG is performed via iterative burr hole creation, and thus relies on efficient, safe, accurate, and consistent drill features. Drill quality has been shown to impact safety and future bone regeneration, with lower drilling speeds and increased pressure leading to improved bone regeneration [6].

Traditionally, orthopedic drills are repurposed for sEEG. Orthopedic drills are capable of drilling at high revolutions per minute (RPM) to provide sufficient torque required for drilling in long bones, typically exceeding the speeds needed for drilling the thinner cranium. Increased RPM can generate increased temperatures which then cause thermal damage to both the patient’s skin and bone and has even led to osteonecrosis [7, 8]. There are no studies comparing the two types of drills for the purpose of sEEG in pediatric patients. The specific aim of this study is to compare immediate intraoperative safety, thermal injury risk, drilling efficiency, and hardware-specific complication rates during burr-hole creation with a conventional orthopedic drill versus a neurosurgical drill. To further investigate the impact of drill design on sEEG burr hole creation safety and efficiency, we identified a retrospective cohort of pediatric patients who underwent sEEG using either a Phasor^®^ drill or a Stryker^®^ drill.

## Methods

### Patient selection

This study was approved by the Ann & Robert H. Lurie Children’s Hospital of Chicago (LCH) Institutional Review Board (IRB #2024–6651) prior to the intuition of this study. A prospective database of all patients undergoing neurosurgery at LCH from January 2021 to present was searched. All sEEG surgeries performed by surgeon (JSR) in the years 2022–2023 were included. Seventeen patients met inclusion criteria. Demographic factors collected included age, sex, race/ethnicity. We also collected clinical data, underlying etiology of DRE, and operative characteristics including laterality, operative duration, bone thickness, and number of electrodes placed. This study was conducted at Ann & Robert H. Lurie Children’s Hospital of Chicago as a retrospective review of existing medical records. The IRB determined the project to be exempt human subjects research in accordance with federal regulations (IRB# 2024–6651). Because the study met criteria for exempt status and involved no contact with participants, informed consent and assent was not required.

### Surgical procedure

Under anesthesia and after appropriate pre-operative antibiotics, sEEG was performed through a standard approach using bone fiducials and frameless stereotactic navigation using the ROSA ONE® Brain (Zimmer Biomet, Warsaw, IN, USA) system to place depth electrodes. Standard workflow and tools have been previously described [1]. Following electrode placement, fiducials are removed, a stitch is placed, and the patients are observed in the epilepsy monitoring unit.

### Drills

**Fig. 1:**
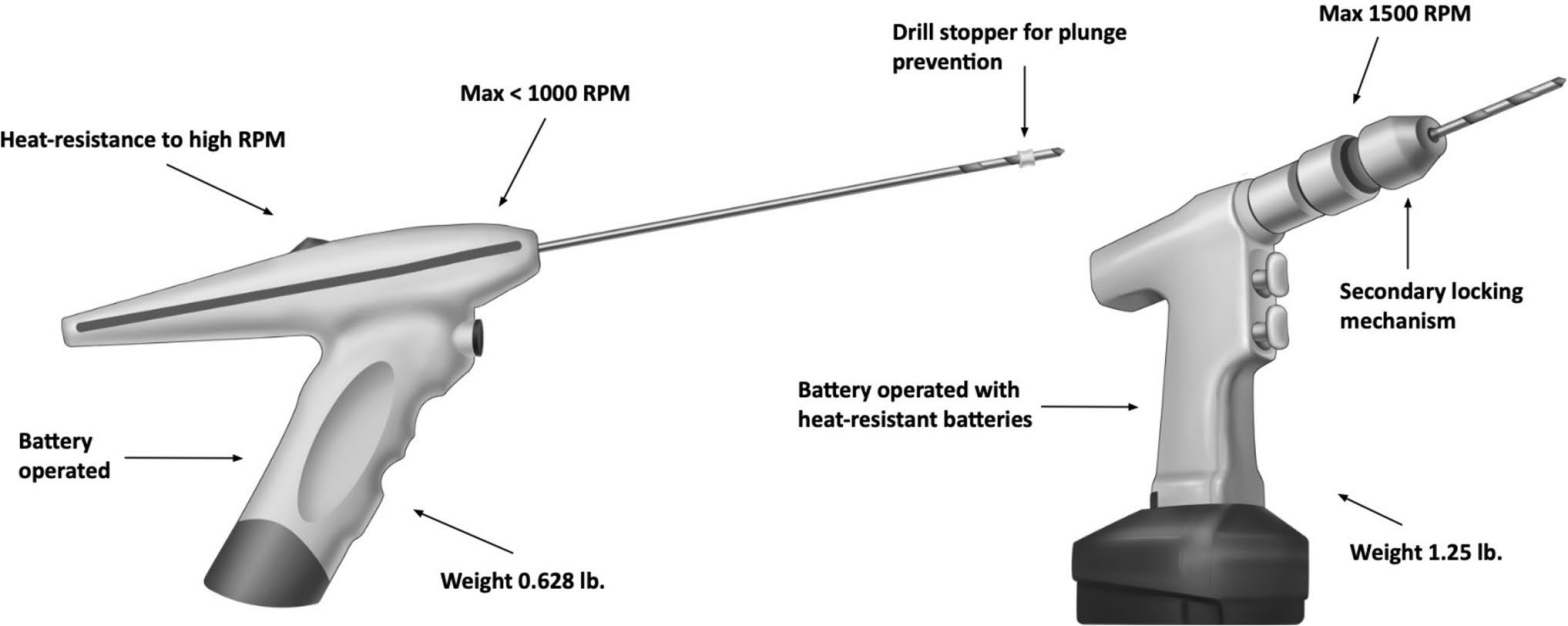
Visual representation of the Phasor^®^ drill compared to the Stryker^®^drill with advantages highlighted. Right, the neurosurgery-tailored drill limits local heating through an RPM regulated system. The disposable drill is battery operated. The drill has a variety of bits that are all adjustable by length and a rotated drill stopper to prevent plunging; Left, the Stryker. ^®^ orthopedic drill is reusable and contains heat-resistant batteries. The drill also contains a secondary locking mechanism and a depth stopper (not pictured)

Two drills are studied: neurosurgery-tailored–Phasor disposable battery-operated drill 3.20-mm diameter, 210-mm usable length (Phasor Health, LLC, Houston, Texas, USA)–or conventional orthopedic–Stryker® System 8 cordless driver (Stryker Instruments, Portage, Michigan, USA) (Fig. 1). The Stryker® drill was utilized for burr hole creation until September 2022, after which the Phasor® drill is used.

### Drill comparison

A cost-analysis was performed based on the differences between cost (monetary request from the hospital) and charge (monetary request from the payors) for each drill. A technical analysis was also performed on each drill including technical specifications (RPM, anti-plunging, automatic stopper) as well as user metrics including the tactile feel of the cortical and cancellous bone, instability while drilling, and drilling efficiency and consistency. Drilling efficiency is measured using subjective drill time per burr hole. Drilling consistency is measured by comparing the drill time at the first burr hole with the drill time at the last burr hole. Operative time is used as an objective marker for overall drilling efficiency. Descriptive analysis was performed in Excel.

### Safety-analysis

Patients are grouped by type of drill used intra-operatively and post-operative drill-related complications are recorded. Complications included burn/wound dehiscence.

## Results

### Demographics and baseline surgical information

Fifteen patients (8 female) are included. A total of seventeen sEEG procedures were performed. Mean age at operation was 13.7 years (range 7–20). DRE was secondary to tumor or tumor resection (n = 7), focal cortical dysplasia (n = 3), lesional (hippocampus, temporal lobe, and parietal lobe; n = 3), and idiopathic (n = 2). Twelve patients were treated with the Phasor® drill, and five patients were treated with the Stryker® drill. Two patients underwent sEEG twice and are represented in both cohorts (Table 1).

**Table 1:** Patient demographics

Case No	Age, Sex	Race, Ethnicity	Etiology of MRE
*Stryker Drill*			
1	20, F	Other, H	Lesional (tumor removal)
2	14, M	White, NH	Lesional (tumor)
3^a^	7, M	Other, H	Lesional (tumor)
4	15, M	White, H	Lesional (FCD)
5^a^	16, F	White, NH	Idiopathic
*Phasor Drill*			
6^a^	7, M	Other, H	Lesional (tumor)
7^a^	16, F	White, NH	Idiopathic
8	16, F	White, NH	Lesional (tumor)
9	14, M	White, NH	Lesional (hippocampus)
10	9, M	White, NH	Lesional (tumor)
11	16, M	Black, NH	Lesional (tumor removal)
12	11, F	White, NH	Lesional (tumor)
13	14, M	White, NH	Lesional (FCD)
14	15, F	White, NH	Lesional (temporal lobe)
15	17, M	Black, NH	Lesional (FCD)
16	11, F	White, NH	Lesional (parietal lobe)
17	14, F	White, NH	Idiopathic

No = Number; F = female; M = male; H = Hispanic; NH = non-Hispanic; MRE = medically refractory epilepsy

^a^Two patients had SEEG twice and are represented in both groups, cases 3 and 6 are the same patient and cases 5 and 7 are the same patient

### Drill comparison

In the Phasor® cohort, two patients underwent unilateral electrode placement, and ten patients underwent bilateral electrode placement. An average of 10.7 ± 1.6 electrodes were placed per patient. In the Stryker® cohort, two patients had unilateral electrode placement, and three patients had bilateral electrode placement. An average of 11 ± 2.9 electrodes were placed per patient. The mean operative duration was 162.9 ± 49 min and 176.4 ± 18.9 min for the Phasor® cohort and Stryker® drill cohort, respectively (Table 2).

**Table 2:** Clinical features

Case No	Laterality	Electrodes Placed (number)	Operative Duration (minutes)
*Stryker Drill*			
1	Unilateral	9	157
2	Unilateral	9	178
3^a^	Bilateral	10	166
4	Unilateral	16	174
5^a^	Bilateral	11	207
*Phasor Drill*			
6^a^	Bilateral	10	135
7^a^	Bilateral	11	92
8	Bilateral	11	164
9	Unilateral	11	166
10	Bilateral	10	210
11	Bilateral	9	85
12	Bilateral	11	160
13	Bilateral	10	169
14	Bilateral	11	168
15	Bilateral	10	208
16	Unilateral	9	136
17	Bilateral	15	262

^a^Two patients had SEEG twice and are represented in both groups, cases 3 and 6 are the same patient and cases 5 and 7 are the same patient

Subjective and objective differences in drills were also evaluated (Tables 3 and 4). Surgeon (JSR) reported good tactile feel of bony layers, drill time per burr hole (< 15 s, without significant change in drilling duration between first and final burr hole in each patient), and coaxial burr hole-bolt alignment (without instability) when using the Phasor® drill. Subjectively, the Stryker® drill is noted to have higher variability with drill time per burr hole and increased drill time duration between the first and last burr hole in a case. Objectively, the Stryker® drill has a max RPM of 1500 and weighs 1.25 lb. The Phasor® drill has a max RPM < 1000 and weight of 0.628 lb. The Phasor® drill cost $398, and the charge was $159. The bits for the Stryker® drill cost $162.36 and the charge was $649.44. Cost remained the same throughout the entire study period. Though acquisition of the Stryker® drill is cheaper for the hospital, the Phasor® drill is four times cheaper for the patient.

**Table 3:** Subjective difference in drills

Conventional orthopedic drill	Neurosurgery-tailored drill
Variable drill time	< 15 s per drill hole
Increased duration in drilling between first and last hole	Consistent duration in drilling between first and last hole
	Coaxial burr hole-bolt alignment without wobbling
	Tactile feel of bony layers

**Table 4:** Objective difference in drills

	Conventional orthopedic drill	Neurosurgery-tailored drill
Max RPM	1500 RPM	< 1000 RPM
Weight	1.25 lb	0.628 lb
Charge	$649.44	$1592
Cost	$162.36	$398

### Safety-Analysis

**Fig. 2:**
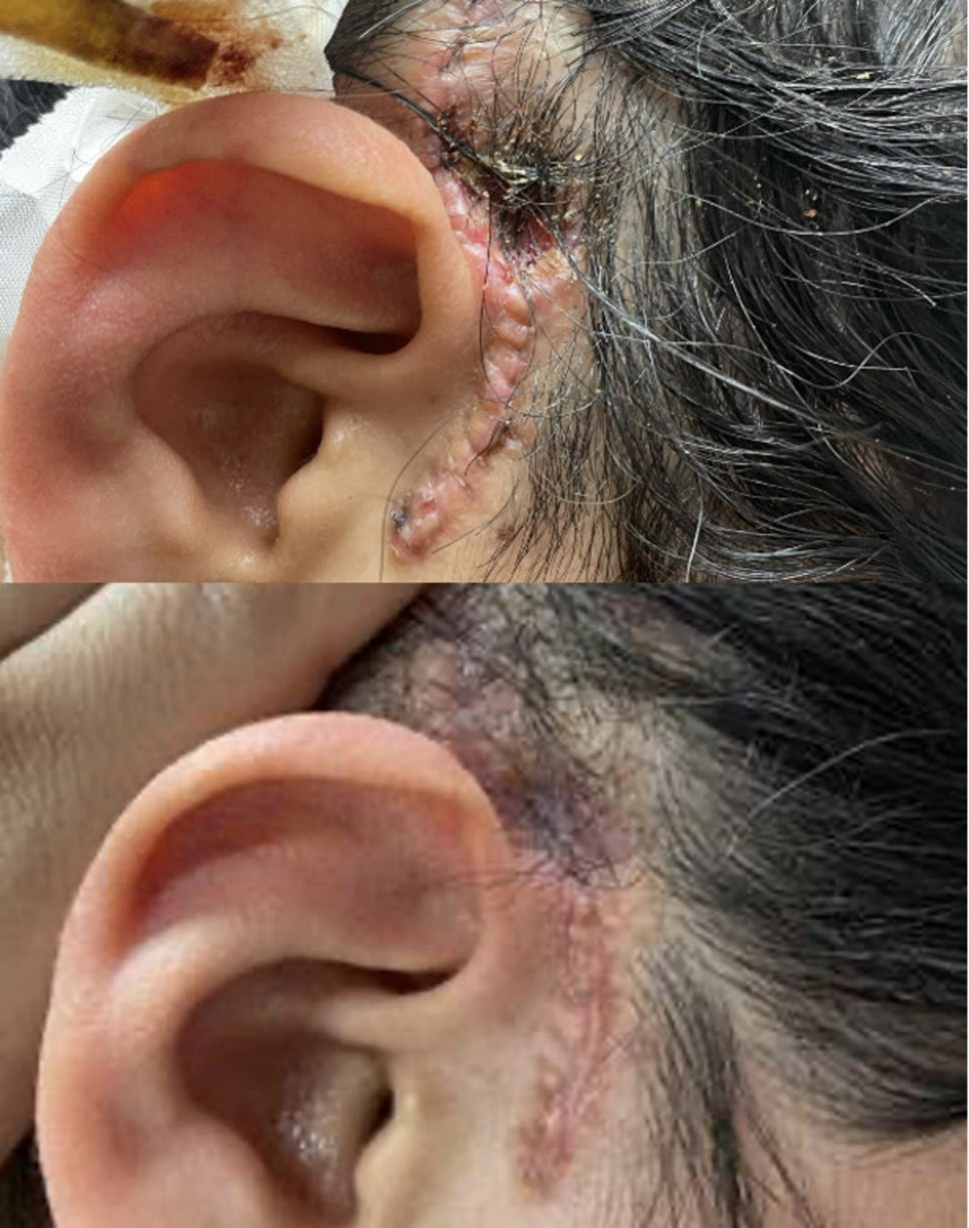
Skin burns incurred from Stryker^®^ drill. Upper, thermal injury with skin necrosis, noted intraoperatively which caused wound dehiscence and infection. Lower, healed wound after three months of daily wound care with MediHoney^®^ and clindamycin

The Phasor® cohort did not experience any complications in bolt migration, dislodgement, or wound issues. One patient (Case 1) in the Stryker® cohort incurred a severe skin burn at the drill site (Fig. 2). This was noted intraoperatively and is attributed to increased drilling time caused by thick cortical bone. This patient experienced wound dehiscence and infection requiring daily wound care and a three-month course of clindamycin until the wound healed.

## Discussion

A Phasor® drill may offer an advantage to both the surgeon and patient. Efficiency and ease of use are improved in the Phasor® cohort compared with the Stryker® cohort. The consistency in drill sharpness and effectiveness when creating burr holes made the Phasor® drill reliable and preferable for sEEG procedures. The lack of complications intraoperatively and perioperatively (0%, 0/12) may make the Phasor® drill favorable for patients as well. The Stryker® drill demonstrates less drilling consistency, increasing drill time throughout the operation, and suboptimal for sEEG efficiency. To our knowledge, this is the first study comparing the use of Phasor® drill to Stryker® drill.

Thermal injury is uncommon in general and is reported as a drill-related complication rate of 1.4% for sEEG [5]. It is unclear if this number represents a per burr hole rate or a per patient rate, although in both cases our small cohort underperformed that metric (1.8% per burr hole, 20% per patient). The skin burn caused by the drill was due to the excessive heat generated from the Stryker® drill, likely exacerbated by power reduction over time and dulling of the drill bit. The thermal injury led to poor wound healing and a subsequent bacterial infection requiring extensive follow-up for wound care. As a result of this incident, our institution transitioned to using the Phasor drill for use in sEEG cases to prevent future adverse events and improve patient safety.

Oral and maxillofacial surgeons and orthopedic surgeons have conducted numerous studies to optimize drill use to decrease operative time and reduce negative outcomes for patients [9–13]. Jamil and colleagues characterized the important parameters for drill optimization as either crucial drilling characteristics (e.g., performance measures such as torque, surface roughness, pullout strength, and temperature) or critical parameters (e.g., drill geometric features, bone-specific parameters, irrigation system, and cutting conditions) [13]. Many studies have reported that excessive drill forces (e.g., torque) can cause increased thrust and temperature leading to perforation, soft tissue damage, and osteonecrosis [14].

Drilling forces are significantly affected by bone type, requiring drill parameters to be tailored appropriately. ^14,15^ Drills that have been optimized for thick cortical bone, such as the femur (orthopedic surgery specific, average width 28.6 ± 2.1), may not be optimized for drilling the neurocranium, where the thickest section is 3.4–7.3 mm in width (parietal bone) [16, 17]. A lower RPM and torque are needed to drill into the much thinner cranium. Higher RPM as found in orthopedic drills causes excess heat generation; in cases like ours, higher RPM does not lead to faster hole creation. Although the Stryker® drill has heat-sensitive batteries to prevent overheating and electrical malfunction of the drill components, it does not dissipate heat generated during surgery [18]. In contrast, the Phasor® drill may temper the local thermal experience via lower RPM with a sharper disposable bit; plunge prevention with a depth stopper was used in both cohorts without any difference [13–19].

Although complications due to sEEG are rare and not entirely preventable, complications due to utilization of an orthopedic drill in a neurosurgical setting are preventable. Using the Phasor® drill in the sEEG setting offers the opportunity to improve patient safety and surgical outcomes by reducing near-miss events. Near-miss events are errors that have the potential to cause harm to a patient but do not by way of chance [20, 21]. These events can be difficult to capture because patients remain unharmed, making them often less visible (e.g., the orthopedic drill frequently overheats because it is optimized for drilling into femur rather than cranium, but the surgeon has not yet burned a patient or caused noticeable osteonecrosis) [22].

Patient safety requires vigilance for near-misses to avoid never-events [23]. Never-events cause serious harm to patients (e.g., the use of the orthopedic drill causes extensive skin burns) [23–25]. Based on this framework, Case 1 is a never-event and cases 2–5 are unrecognized near-miss events. In the case of sEEG, a procedure with an Even in cases like sEEG, with relatively low complication rates, patients may be safer by using a neurosurgical drill. The Phasor® drill offered ergonomic advantages due to its lightweight nature, reliable tactile feedback, and drill bit stability [19].

Our study is limited by the small sample size, single center, single user, and subjective reporting of many metrics. The comparison groups were unbalanced due to our institutional replacement of Stryker® orthopedic drills with Phasor® drills for sEEG cases following the described complication. Differences noted are not statistically significant due to the small sample size. Despite these limitations, patient safety was the motivation for efforts to optimize drill use for sEEG. There has not been another adverse event since the switch to a Phasor® drill, although sEEG complications are rare and the follow-up was constrained to intraoperative events. Future studies may report per burr hole complication rates and focus on optimizing drill performance while balancing patient safety and operative efficiency metrics.

In conclusion, there were no complications using a drill optimized for drilling the neurocranium, while 20% of our small cohort had a thermal induced complication from a drill optimized for orthopedic surgery. SEEG hardware-related complications may be under reported and additional multi-institutional studies with larger sample sizes may further reveal drill-dependent differences in pediatric sEEG morbidity.

## Data Availability

All data supporting the findings of this study are available within the manuscript.
